# Why So Serious? An Attempt to Mitigate the Short-Term Harmful Effects of the Film Joker on Prejudice toward People with Mental Illness

**DOI:** 10.3390/bs12100384

**Published:** 2022-10-07

**Authors:** John William Poulgrain, Niquaila May Bremner, Hannah Zimmerman, Chia-Wei Jao, Taylor Winter, Benjamin Charles Riordan, Boris Bizumic, John Hunter, Damian Scarf

**Affiliations:** 1 Department of Psychology, University of Otago, Dunedin 9054, New Zealand; 2 Department of Psychology, Victoria University of Wellington, Wellington 6140, New Zealand; 3 Centre for Alcohol Policy Research, La Trobe University, Melbourne 3550, Australia; 4 Research School of Psychology, Australian National University, Canberra 2601, Australia

**Keywords:** mental illness, stigma, prejudice

## Abstract

The media perpetuates many harmful stereotypes about people with mental illness. In two studies, we demonstrate the impact of negative media portrayals of mental illness on prejudice and attempt to mitigate these negative effects. Specifically, in Study 1, participants watched the movie *Joker*, a recent film which associates mental illness with violent behavior, or a control film (*Terminator*). Participants completed the Prejudice towards People with Mental Illness (PPMI) scale before and after viewing their respective films. The PPMI consists of four dimensions: fear/avoidance (i.e., wanting to avoid people with mental illness), malevolence (i.e., viewing people with mental illness as inferior), authoritarianism (i.e., preference for control over people with mental illness), and unpredictability (i.e., the behavior of people with mental illness is unreliable). We hypothesized that participants who watched the film *Joker* would display an increase in their fear/avoidance of people with mental illness and their support for authoritarian approaches to their treatment. Consistent with these hypotheses, participants who viewed *Joker* displayed a significant increase in the fear/avoidance and authoritarian subscales of the PPMI, relative to participants that watched *Terminator*. In Study 2, in an attempt to mitigate the impact of *Joker* on prejudice towards people with mental illness, directly after the film we displayed educational and counter-stereotypical statements on-screen that challenged the view that people with mental illness are violent. A control group viewed *Joker* without these statements. Identical to Study 1, all participants completed the PPMI scale before and after viewing the film. We hypothesized that participants who viewed *Joker* with the statements would display lower prejudice relative to the control condition. Unfortunately, participants in the experimental and control conditions displayed a comparable increase in prejudice. Together, these studies confirm the negative effect of media portrayals of mental illness (as depicted in *Joker*) and demonstrate that these effects are not easily mitigated.

## 1. Introduction

Goffman [1] defined stigma as a label attached to a stereotype, connecting the stigmatized person to an undesirable characteristic or trait. For people with mental illness, these stereotypes can range from being intellectually brilliant to profoundly violent. Largely, these stereotypes are derived from popular media (e.g., news, movies, music). Indeed, the media is the primary avenue through which most people learn about mental illness [2,3]. As news media is purportedly non-fiction, one would expect that it provides the public with an accurate and objective representation of people with mental illness. Unfortunately, this is rarely the case [2,4]. For example, Corrigan, Watson, Gracia, Slopen, Rasinski and Hall [4] examined a large sample of newsprint stories that focused on mental illness or mental health and coded them into one of four categories: dangerousness, blame, treatment/recovery, and advocacy. From a final sample of 3553 news stories, the most common themes were related to dangerousness. Assessing change over time, McGinty et al. [5] analyzed news media coverage between 1995 and 2014 in the United States. Although there was a decrease in the total number of stories over time, the stories that included mental illness still predominantly focused on violence. Stereotypical news coverage of mental illness is not restricted to the United States, with similar findings reported in Japan [6], Canada [7] and Aotearoa, New Zealand [2].

Beyond news media, stereotypical portrayals of mental illness have a long history in film. For example, in the 1975 film *One Flew Over the Cuckoo’s Nest*, Jack Nicholson plays Randle McMurphy, a character who feigns insanity to avoid prison. Unfortunately, the character is placed in a psychiatric hospital, deemed dangerous and receives a frontal lobotomy due to his rebellious behavior. More recent films have tended to depict characters experiencing, rather than feigning, symptoms of severe mental illness. For example, Owen [8] analyzed characters from 41 films that depicted or displayed symptoms of schizophrenia, reporting that many of the characters were violent towards themselves and others.

Several studies have investigated how viewing films depicting mental illness influences prejudice. For example, Domino [9] investigated the impact of watching *One Flew over the Cuckoo’s Nest* on attitudes towards mental health professionals, hospitals and facilities and people with mental illness. Participants’ attitudes towards all three areas were more negative after watching the film. More recently, Perciful and Meyer [10] examined the impact of four films that varied in their portrayal of mental illness and likability (i.e., emotional tone). Broadly speaking, the films were rated as inaccurate but likable (*Me, Myself and Irene*), inaccurate and fear-based (*Donnie Darko*), accurate and educational (*The Brush, the Pen and Recovery*) and neutral (a control film). Participants who viewed the fear-based film displayed a desire for greater social distance from people with mental illness, compared to participants who watched the other three films. Promisingly, social distance scores for participants who viewed the accurate film were significantly *lower* than those who viewed the inaccurate films, suggesting participants had fewer stigmatizing attitudes when they had viewed an accurate portrayal of mental illness. As this latter finding suggests, the influence of film media is not always negative. Indeed, Thonon, et al. [11] examined whether a documentary film (*Radio Schizo*) reduced negative attitudes towards people with schizophrenia. The documentary followed five young people diagnosed with schizophrenia, with the young people at a range of stages regarding symptom severity and living conditions. After viewing the film, participants displayed a reduction in their view that people with mental illness are dangerous.

Reducing Mental Illness Stigma

A large number of studies have investigated whether mental illness stigma can be reduced via mass-media campaigns and more targeted interventions [12,13,14,15,16]. Approaches to reducing mental illness stigma have tended to focus on contact and education. Contact-based approaches draw on social psychology theory and research, suggesting that contact with out-group members reduces the anxiety and fear associated with unknown groups [17]. Although the literature on contact has primarily focused on racial and ethnic groups, several studies have investigated whether direct (i.e., interpersonal) or indirect contact (e.g., video-based) can reduce mental illness stigma [15,18]. For example, Corrigan et al. [19] had college students listen to a 10-minute presentation given by an individual with a history of severe mental illness. As part of the presentation, the individual not only outlined their history of mental illness (e.g., symptoms, experiences of hospitalization) but also noted that, although they were not symptom-free, they were working and living independently. Further, to enhance the effects of contact, the 10-minute presentation was followed by a 5-minute discussion, providing a context for the students to interact with the individual. Results demonstrated that contact improved attributions about the controllability and stability of people with mental illness [19].

Similar to contact, education-based approaches challenge myths regarding people with mental illness. Although contact does this through interpersonal interaction (e.g., meeting an individual with mental illness who is stable and working), education takes a more direct approach, specifying the myths associated with mental illness and countering them with evidence. For example, Finkelstein, et al. [20] presented students with a computer-based program that provided information on mental illness (e.g., incidence, causes, treatment) and challenged several myths regarding mental illness (e.g., people with mental illness are dangerous). The program not only significantly increased students’ knowledge regarding mental illness but also significantly reduced mental illness stigma.

## 2. Mitigating the Negative Effects of Film

Despite clear evidence that films can increase mental illness stigma, there is little evidence that the depiction of mental illness in film has improved over time. Given this, an important question is whether the negative effects of viewing a film can be mitigated or reduced. In one of the few studies to address this question, Wahl and Lefkowits [21] had participants watch the 1986 film *Murder: By Reason of Insanity*, which told the true story of a man who murdered his wife while out on a day pass from a psychiatric hospital. Before and after the film, a narrator read an on-screen statement that noted the violent behavior depicted in the film was “not intended to be a general reflection of the mentally ill” and that people with mental illness “are not more prone to violence than the non-mentally disordered”. Unfortunately, the educational information did not mitigate the adverse effects of viewing the film, with participants in the education condition displaying comparable increases in prejudice (e.g., people with a mental illness are dangerous) to those who watched the film without the educational statements.

More recently, Ritterfeld and Jin [22] investigated whether a trailer played before or after the movie *Angel Baby* could mitigate the movie’s negative effects. *Angel Baby* was released in 1995 and tells the story of Harry and Kate, two people with schizophrenia that meet each other during a group therapy session. They start a romantic relationship and, upon finding Kate is pregnant, discontinue their medication. The film follows the negative impact of them discontinuing their medication. Ritterfeld and Jin [22] developed six different types of trailers that varied in their focus on the movie (i.e., comments about specific characters vs. general information about schizophrenia) and whether the person speaking was a patient with mental illness or a psychiatrist. Promisingly, although the movie alone increased mental illness stigma, pairing the movie with one of the six trailers significantly reduced mental illness stigma. Moreover, this reduction was more marked when the trailer followed, rather than preceded, the film. 

## 3. Current Study

Building on the work of Wahl and Lefkowits [21] and Ritterfeld and Jin [22], the current study attempts to mitigate the negative effect of the film *Joker*. *Joker* was released in 2019 and became the first R-rated film to break 1 billion dollars at the box office. In addition to its popularity, our interest in *Joker* is a result of the movie 1) portraying its namesake character as having a mental illness and 2) following a narrative in which, following the character stopping his medication, he carries out a campaign of violence (including murder). Thus, the film does what many films before it have done: it associates mental illness with acts of violence. Previously, we have reported that watching *Joker* increases prejudice towards people with mental illness [23]. Here, in Study 1, we reanalyze these data and investigate whether *Joker* impacts specific dimensions of prejudice. Specifically, while Scarf, Zimmerman, Winter, Boden, Graham, Riordan and Hunter [23] assessed the impact of *Joker* on the overall score on Kenny, Bizumic and Griffiths’ [24] Prejudice towards People with Mental Illness (PPMI) scale, we investigate the impact of *Joker* on the PPMI’s four dimensions: fear/avoidance (i.e., wanting to avoid people with mental illness), malevolence (i.e., viewing people with mental illness as inferior), authoritarianism (i.e., preference for control over people with mental illness) and unpredictability (i.e., the behavior of people with mental illness is unreliable). Given the themes in *Joker*, we hypothesized that watching the film would lead to significant increases in participants’ fear/avoidance of people with mental illness and their support for authoritarian approaches to the treatment of people with mental illness (e.g., enforced treatment).

Building on Study 1, in Study 2 we attempt to mitigate the negative impact of *Joker* by presenting messages that challenge one of the main themes of the film (i.e., that people with mental illness are dangerous). Specifically, two messages were presented on the screen directly after the film (i.e., “90–95% of people diagnosed with schizophrenia or bipolar disorder do not commit any violent crimes” and “People with mental illness are over 10 times more likely to be the victim of violent crime than the general population”). Study 2 had two hypotheses. First, that participants in both the educational and control (i.e., no educational messages) conditions would display an increase in prejudice. Second, that the increase in prejudice would be less marked for participants in the educational condition, relative to participants in the control condition. 

## 4. Study 1

### 4.1. Method

#### 4.1.1. Participants

As noted above, Study 1 used data from Scarf, Zimmerman, Winter, Boden, Graham, Riordan and Hunter [23]. Demographic information is provided in Table 1. Participants were members of the general public, recruited through poster advertising and online advertising on websites such as Facebook. The only inclusion criterion was that participants were required to be aged 18 or above. Participants were compensated with $20 to cover travel costs to and from the theatre. The current study was reviewed and approved by the University of Otago Human Ethics Committee (19/108).

#### 4.1.2. Procedure

Participants were asked to complete survey 1 online. Survey 1 opened with an information and consent form. Once participants consented to take part, they were presented with demographic questions, followed by several questionnaires. Specifically, participants completed the contact and prejudice measures outlined below, in addition to the social/stress and heredity/biology subscales of the Mental Illness Attribution Questionnaire [25], the Very Short Authoritarianism Scale [26], the Social Dominance Orientation scale [27], the empathic concern subscale of the Interpersonal Reactivity Index [28] and the beliefs for others subscale of the Justice Beliefs scale [29].

The movie screening occurred 2 to 3 weeks after participants completed survey 1, with participants attending a film screening at a local theatre in Dunedin, Aotearoa New Zealand. On arrival at the theatre, participants were randomly allocated to one of two large (>100 seats) theatres. In one theatre, participants viewed *Joker*, while in the other, they simultaneously viewed *Terminator: Dark Fate*. After viewing the film, participants completed survey 2 in the theatre as a paper and pencil survey. Except for the demographic information and contact scale, all scales from survey 1 were repeated in survey 2. 

#### 4.1.3. Films

*Joker. Joker* was released in 2019. The film was rated R due to the language in the film, brief sexual images and violence. The protagonist in the film, Arthur Fleck, takes medication and displays symptoms typically associated with schizophrenia, including both hallucinations and grandiose delusions. It is important to note that no specific diagnosis of schizophrenia is given in the film. Over the course of the film, Arthur loses his job and the help of his psychologist and stops taking his medication due to a lack of funding. Following this series of events, he becomes violent and increasingly unpredictable, brutally killing a co-worker, three subway passengers and a talk show host. The film ends with his implied killing of a prison psychologist, the final film shot being his walking and dancing along a corridor, leaving bloody footsteps in his wake.

*Terminator*. *Terminator: Dark Fate* was released in 2019. The film was rated R due to the language in the film, brief nudity, and violence. It is the sixth film in the Terminator franchise and provides a sequel to *The Terminator* (1984) and *Terminator 2* (1991). The plot is similar to that of *Terminator 2*, with a terminator sent back in time to rewrite history and kill a character that challenges Skynet (an artificial intelligence) at a future time point. The reason for selecting *Terminator 2* is that it was also currently showing and, importantly, also contained violence. 

#### 4.1.4. Measures

*Demographic*. Participants completed a questionnaire on general demographic information such as age, ethnicity and gender.

*Contact*. Contact with mental illness was assessed using six items. Specifically, participants were asked whether they currently or have ever: had a mental illness, lived with someone with a mental illness, worked with someone with a mental illness, had a neighbor with a mental illness, had a close friend with a mental illness, had a family member with a mental illness. The contact measure was only included in survey 1.

*Prejudice*. Mental illness prejudice was assessed using the PPMI [24]. As noted above the PPMI has four subscales measuring fear/avoidance (e.g., “I am not scared of people with mental illness”), malevolence (e.g., “People who become mentally ill are not failures in life”), authoritarianism (“People who are mentally ill should be forced to have treatment”) and unpredictability (e.g., “People with mental illness often do unexpected things”). Responses were scored on a 7-point Likert scale (1 = *strongly disagree* to 7 = *strongly agree*). The subscales display adequate reliability at both Time 1 (Fear/avoidance: α = 0.832; Malevolence: α = 0.688; Authoritarianism: α = 0.717; Unpredictability: α = 0.765) and Time 2 (Fear/avoidance: α = 0.889; Malevolence: α = 0.737; Authoritarianism: α = 0.846; Unpredictability: α = 0.845). The PPMI was included in both survey 1 and survey 2.

#### 4.1.5. Data Analysis

Each of the four subscales was submitted to a repeated measures Analysis of Variance (ANOVA), with Time (2: Before, After) as a within-participant factor and Film (2: *Joker*, *Terminator*) as a between-participant factor. Contact was added as a covariate.

### 4.2. Results

**Figure 1:**
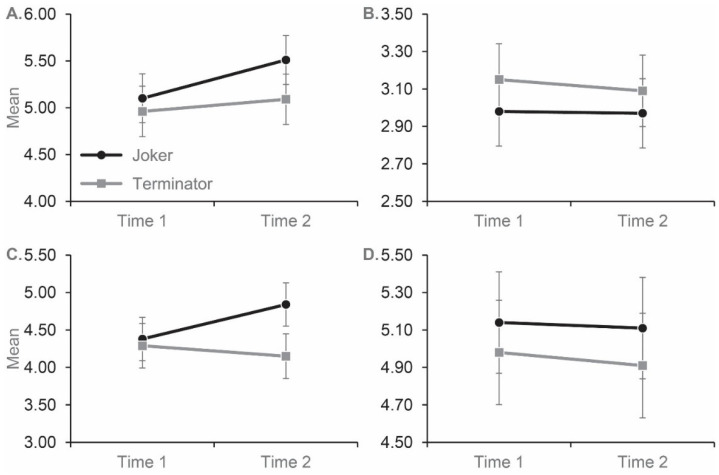
Mean scores for participant in the experimental (*Joker*) and control (*Terminator*) conditions on the fear/avoidance (**A**), malevolence (**B**), authoritarianism (**C**) and unpredictability (**D**) subscales of the PPMI. The subscales were completed before (Time 1) and after (Time 2) each film. A total of 81 participants viewed *Joker*, and 80 viewed *Terminator*. Error bars represent ± standard error of the mean.

For fear/avoidance and authoritarianism, and relevant to the hypothesis that prejudice would be higher for those who watched *Joker*, we identified a significant Time by Film interaction (Fear/avoidance: *F*(1, 158) = 7.532, *p* = 0.007, *partial eta^2^* = 0.046; Authoritarianism: *F*(1, 158) = 24.851, *p* < 0.001, *partial eta^2^* = 0.136; Figure 1). To investigate the interaction, we conducted post-hoc paired-sample t-tests. Participants who watched *Joker* displayed an increase in fear/avoidance and authoritarianism from Time 1 to Time 2 (Fear/avoidance: *t*(79) = 5.20, *p* < 0.001, *Cohen’s d* = 0.581; Authoritarianism: *t*(79) = 4.86, *p* < 0.001, *Cohen’s d* = 0.543), while those who watched *Terminator* displayed no significant change on neither fear/avoidance nor authoritarianism (Fear/avoidance: *t*(80) = 1.83, *p* = 0.071, *Cohen’s d* = 0.203; Authoritarianism: *t*(80) = 1.97, *p* = 0.052, *Cohen’s d* = 0.219).

In contrast to fear/avoidance and authoritarianism, for malevolence and unpredictability there was no main effect of Film (Malevolence: Film, *F*(1, 158) = 2.56, *p* = 0.111, *partial eta^2^* = 0.016; Unpredictability: *F*(1, 158) = 1.90, *p* = 0.170, *partial eta^2^* = 0.012) or Time by Film interaction (Malevolence: *F*(1, 158) = 0.367, *p* = 0.545, *partial eta^2^* = 0.002; Unpredictability: *F*(1, 158) = 0.108, *p* = 0.742, *partial eta^2^* = 0.001). There was also no main effect of Time for either measure (Malevolence: *F*(1, 158) = 0.029, *p* = 0.865, *partial eta^2^* = 0.000; Unpredictability: *F*(1, 158) = 0.944, *p* = 0.333, *partial eta^2^* = 0.006).

### 4.3. Discussion

Study 1 hypothesized that participants who watched the film *Joker* would display an increase in their fear/avoidance of people with mental illness and their support for authoritarian approaches to their treatment. Consistent with these hypotheses, participants who viewed *Joker* displayed a significant increase in the fear/avoidance and authoritarian subscales of the PPMI [24]. In contrast, there was no evidence that viewing *Joker* increased participants’ views regarding malevolence or their belief that people with mental illness behave in an unpredictable manner. These findings are broadly consistent with the themes displayed in the movie. That is, following the discontinuation of his medication, Joaquin Phoenix’s character is depicted carrying out a range of serious and violent crimes. Given this, it is not surprising people become more fearful of people with mental illness and, as a result, were more supportive of policies such as enforced treatment.

With respect to malevolence, the film’s sympathetic depiction of Arthur’s life is contrasting to the view that he is avoiding the difficulties of everyday life. If anything, Arthur is depicted as experiencing difficulties over and above that which we could consider normal, including an extremely difficult upbringing (e.g., a parent with severe mental illness) and being treated poorly as an adult. Thus, the film is more likely to generate sympathy towards people with mental illness, which may, in turn, diffuse any potential feelings and beliefs related to malevolence. The way Arthur is treated in the film also relates to the null finding regarding the unpredictability subscale, as any violent behavior displayed in the film is predictable, in the sense that it is (1) a direct response to either being humiliated or physically assaulted and (2) is relatively consistent across the course of the film. 

As *Joker* makes clear, modern-day films still associate mental illness with violence. An important question is whether the negative impact of films can be mitigated. As noted above, typically, as part of educational interventions, some studies have directly addressed the stereotypes we hold about people with a mental illness (e.g., that they are violent). For example, Corrigan et al. [30] contrasted the myth that “persons with serious mental illness like schizophrenia are violent and should be avoided” with the fact that “most persons with serious mental illness are no more violent than the average citizen”. Participants presented with this information displayed improvement in their attitude towards people with mental illness.

Therefore, in Study 2 we utilize this myth-challenging approach by displaying educational messages at the end of *Joker*. Specifically, we focused on including messages to counteract the increase in fear of those with a mental illness. That is, participants watched a version of *Joker* in which, immediately after the movie but before the credits, the following statements were displayed: “90–95% of people diagnosed with schizophrenia or bipolar disorder do not commit any violent crimes” and “People with mental illness are over 10 times more likely to be the victim of violent crime than the general population”. Both statements were displayed for 12 seconds. We also included a control condition that, identical to Study 1, simply involved participants watching *Joker* without the educational statements. Study 2 had two hypotheses. First, that participants in both the educational and control conditions would display an increase in prejudice. Second, that when directly compared, the increase in prejudice would be less marked for participants in the educational condition, relative to participants in the control condition.

## 5. Study 2

### 5.1. Method

#### 5.1.1. Participants

One-hundred-and-seventy-one people participated in Study 2. Demographic information is provided in Table 1. All participants were undergraduate students at the University of Otago recruited through the University of Otago Psychology Research Participation program and advertising in the Department of Psychology. The only inclusion criterion was that participants were required to be aged 18 or above. Participants received either course credit or $30 for participating. Due to the nature of the study, full completion of all scales used was not mandatory, and therefore, participant numbers vary slightly depending on the particular scale. Specifically, a single participant did not complete the Authoritarianism subscale of the PPMI. The current study was reviewed and approved by the University of Otago Human Ethics Committee (19/108).

#### 5.1.2. Procedure

Participants were asked to complete survey 1 online. Survey 1 opened with an information and consent form. Once participants consented to take part, they were presented with demographic questions, followed by several questionnaires. Specifically, participants completed the contact and prejudice measures outlined below, in addition to the social/stress and heredity/biology subscales of the Mental Illness Attribution Questionnaire [25], the Very Short Authoritarianism Scale [26], the Social Dominance Orientation scale [27], the empathic concern subscale of the Interpersonal Reactivity Index [28] and the beliefs for others subscale of the Justice Beliefs scale [29]. 

The movie screening occurred at least a day after participants completed survey 1, with participants attending a film screening at a university lecture theatre. On arrival at the theatre, participants were randomly allocated to one of two large (>100 seats) theatres. In one theatre, participants viewed *Joker* without the educational messages, while in the other participants simultaneously viewed *Joker* with the educational messages. After viewing their respective film, participants completed survey 2 in the theatre, in the form of a paper and pencil survey. With the exception of the demographic information and contact scale, all scales from survey 1 were repeated in survey 2. 

#### 5.1.3. Measures

*Demographic*. Participants completed a questionnaire on general demographic information such as age, ethnicity and gender.

*Previously seen Joker.* Study 2 was conducted well after *Joker* had moved from screening only at movie theatres to being available to buy/stream. Given this, participants were asked whether or not they had previously seen *Joker*, prior to participating in Study 2. 

*Contact*. Contact with mental illness was assessed using six items as in Study 1. The contact measure was only included in survey 1.

*Prejudice*. Identical to Study 1, mental illness prejudice was assessed using the PPMI [24]. The subscales display adequate reliability at both Time 1 (Fear/avoidance: α = 0.686; Malevolence: α = 0.697; Authoritarianism: α = 0.654; Unpredictability: α = 0.753) and Time 2 (Fear/avoidance: α = 0.884; Malevolence: α = 0.762; Authoritarianism: α = 0.782; Unpredictability: α = 0.828). The PPMI was included in both survey 1 and survey 2.

#### 5.1.4. Data Analysis

The data were submitted to a repeated measures ANOVA, with Time (2: Before, After) as a within-participant factor and Condition (2: *Joker*, *Joker* edited) and whether participants had previously seen *Joker* (2: Seen, not seen) as between-participant factors. Contact was added as a covariate.

### 5.2. Results

**Figure 2:**
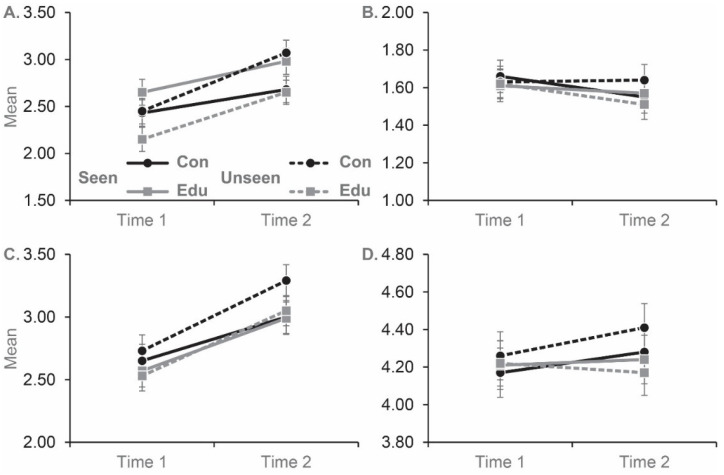
Mean scores for participants in the control (i.e., *Joker* without educational statements) and education (i.e., *Joker* with educational statements) conditions on the fear/avoidance (**A**), malevolence (**B**), authoritarianism (**C**) and unpredictability (**D**) subscales of the PPMI. The subscales were completed before (Time 1) and after (Time 2) each film. A total of 80 participants viewed the control version of *Joker*, and 91 viewed *Joker* with the educational statements. ‘Seen’ refers to participants that had previously seen *Joker*, ‘Unseen’ refers to participants that had not previously seen *Joker*. Error bars represent ± standard error of the mean.

A total of 78 participants (45.6%) had seen *Joker* previously. Replicating Study 1, there was a main effect of time for fear/avoidance, *F*(1, 167) = 52.053, *p* < 0.001, *partial eta^2^* = 0.238, and authoritarianism, *F*(1, 166) = 67.288, *p* < 0.001, *partial eta^2^* = 0.288, but no effect of time for malevolence, *F*(1, 167) = 3.842, *p* = 0.052, *partial eta^2^* = 0.022, or unpredictability, *F*(1, 167) = 1.337, *p* = 0.249, *partial eta^2^* = 0.008 (Figure 2). This implies that for fear/avoidance and authoritarianism, responses were higher post-movie, than pre-movie.

We found no support for our second hypothesis that the edited version of *Joker* would mitigate increases in prejudice when compared to the unedited version of *Joker*. The second hypothesis was tested via a condition by time interaction (Fear/avoidance, *F*(1, 167) = 0.668, *p* = 0.415, *partial eta^2^* = 0.004; authoritarianism, *F*(1, 166) = 0.018, *p* = 0.895, *partial eta^2^* = 0.000; malevolence, *F*(1, 167) = 0.126, *p* = 0.723, *partial eta^2^* = 0.001; unpredictability, *F*(1, 167) = 1.624, *p* = 0.204, *partial eta^2^* = 0.010).

All other main and interaction effects were not significant (all *ps* > 0.202), with the exception of a significant Time by Seen interaction, *F*(1, 167) = 5.302, *p* = 0.023, *partial eta^2^* = 0.031, for the Fear/avoidance subscale. To investigate the Time by Seen interaction further, we conducted t-tests comparing participants who had seen *Joker*, to those who had not seen *Joker*, at Time 1 and Time 2. Participants that had previously seen *Joker* displayed significantly higher levels of fear/avoidance at Time 1, *t*(169) = 2.158, *p* = 0.032, *Cohen’s d* = 0.331, but there was no significant difference between groups at Time 2, *t*(169) = 0.654, *p* = 0.948, *Cohen’s d* = 0.001. These findings suggest that participants that had previously seen *Joker* started the experiment with a higher level of fear/avoidance of people with a mental illness. 

### 5.3. Discussion

Study 2 had two hypotheses. First, that participants in both conditions would display an increase in prejudice. Second, that when directly compared, the increase in prejudice would be less marked for participants in the education condition, relative to participants in the control condition. Our results were mixed. Although we replicated our findings from Study 1, demonstrating that watching *Joker* increased fear/avoidance and authoritarianism, but not malevolence or unpredictability, we found no evidence that the brief educational messages attenuated the negative effects of watching *Joker*.

Interestingly, participants that had previously seen *Joker* reported a higher level of fear/avoidance than participants that had not previously watched the film. There are two potential explanations for this finding. One possibility is that the impact of *Joker* on fear/avoidance of people with mental illness is maintained over time, with the current study revealing the increased level of prejudice participants have held since first watching *Joker*. Alternatively, given participants were informed that they would be watching *Joker* before completing the baseline survey, it is possible providing participants with this information resulted in participants recalling scenes from *Joker*, leading to a transitory increase in mental illness prejudice.

## 6. General Discussion

In Study 1, participants who watched *Joker* displayed a significant increase in their reported fear/avoidance of people with a mental illness and support for an authoritarian approach to their treatment, while participants who watched *Terminator* displayed no such changes. In an attempt to attenuate the negative effect of watching *Joker*, in Study 2, we had participants watch an edited version of *Joker* with two educational statements (e.g., “90–95% of people diagnosed with schizophrenia or bipolar disorder do not commit any violent crimes”) that were displayed immediately after the movie ended, but before the closing credits. Unfortunately, the educational statements had no effect, with participants in both the educational and control conditions displaying a comparable increase in fear/avoidance and authoritarianism.

Our inability to mitigate the negative effect of *Joker* on mental illness prejudice is consistent with Wahl and Lefkowits’ [21] research using the film *Murder: By Reason of Insanity*, but not with Ritterfeld and Jin’s [22] promising results with the film *Angel Baby*. A number of explanations could explain this pattern of findings. For example, *Murder: By Reason of Insanity* and *Joker* are much more explicit with regard to linking mental illness and violence. In contrast, *Angel Baby* has a more complex narrative, with the love story between the two main characters the primary focus of the film. Thus, the significant findings reported by Ritterfeld and Jin [22] may reflect the easier task of mitigating the effects of a film that provides a somewhat balanced view of mental illness. A second important difference is that Ritterfeld and Jin [22] had their trailer narrated by either a person with a mental illness or a psychiatrist, both of which could be important for stigma reduction. Indeed, having a person with mental illness as the narrator is a form of contact, eliciting empathy and potentially providing a counter-stereotypical example of a person with a mental illness. Similar to counter-stereotypical examples, having a psychiatrist as the narrator likely leads people to put more weight on the information included in the trailer, such as information around most people with a mental illness being functioning members of society. 

The current study is not without limitations. First, in the absence of a long-term follow-up, it is not clear whether the increase in prejudice displayed by participants that watched *Joker* is maintained over time. Second, we did not measure actual behavior towards people with mental illness. While participants’ responses to the PPMI provide an estimate of how people may behave, they may be influenced by social-desirability bias [31]. It is important to note, however, that Kenny, Bizumic and Griffiths [24] did not observe any relationship between the PPMI subscales and the short form of the Marlowe-Crowne social desirability scale [32]. Third, in Study 2, we did not assess whether participants paid attention to the educational messages. Finally, we focused on a single movie (i.e., *Joker*). Future studies should investigate how other movies, both old and new, influence the PPMI subscales. 

## 7. Conclusions

Viewing *Joker* increased both fear/avoidance of those with mental illness and support for authoritarian approaches to their treatment. Unfortunately, we were unable to combat these negative effects with brief educational messages. With respect to the title of the current article, *"Why so serious?"* is a line spoken by the Joker in an earlier movie, *The Dark Knight*. One might level that line at the authors of the current study, arguing that *Joker* is simply a work of fiction and nothing to be concerned about. What this view ignores, however, is the profound impact that stigma, and the prejudice therein, has on those suffering from a mental illness.

## Figures and Tables

**Table 1:** Sample demographic information for Study 1 and Study 2.

Variable		Study 1	Study 2
Mean Age (SD)		30.1 (10.2)	20.0 (2.47)
Gender	Male	37.3% (*n* = 60)	17.5% (*n* = 30)
	Female	62.7% (*n* = 101)	81.3% (*n* = 139)
	Other	-	1.2% (*n* = 2)
Ethnicity	New Zealand European	50.3% (*n* = 81)	65.5% (*n* = 112)
	Other European	5.0% (*n* = 8)	2.3% (*n* = 4)
	Māori	5.0% (*n* = 8)	7.0% (*n* = 12)
	Indian	3.1% (*n* = 5)	4.1% (*n* = 7)
	Asian	28% (*n* = 45)	9.4% (*n* = 16)
	Middle Eastern	2.5% (*n* = 4)	1.2% (*n* = 2)
	Pacific Islander	1.9% (*n* = 3)	2.9% (*n* = 5)
	Other	4.3% (*n* = 7)	7.6% (*n* = 13)
Education	High School Level 1 (Year 11)	3.7% (*n* = 6)	-
	High School Level 2 (Year 12)	6.2% (*n* = 10)	3.5% (*n* = 6)
	High School Level 3 (Year 13)	24.8% (*n* = 40)	88.9% (*n* = 152)
	Polytechnic Diploma/Qualification	19.3% (*n* = 31)	0.6% (*n* = 1)
	University Bachelor’s Degree	26.7% (*n* = 43)	7.0% (*n* = 12)
	Honours Degree	7.5% (*n* = 12)	-
	Postgraduate Degree	11.8% (*n* = 19)	-

## Data Availability

The data presented in this study are available on request from the corresponding author.

## References

[1] Goffman E. (1963). Stigma: Notes on the Management of Spoiled Identity.

[2] Coverdale J., Nairn R., Claasen D. (2002). Depictions of mental illness in print media: A prospective national sample. Aust. N. Z. J. Psychiatry.

[3] Reavley N.J., Cvetkovski S., Jorm A.F. (2011). Sources of information about mental health and links to help seeking: Findings from the 2007 Australian National Survey of Mental Health and Wellbeing. Soc. Psychiatry.

[4] Corrigan P.W., Watson A.C., Gracia G., Slopen N., Rasinski K., Hall L.L. (2005). Newspaper stories as measures of structural stigma. Psychiatr. Serv..

[5] McGinty E.E., Kennedy-Hendricks A., Choksy S., Barry C.L. (2016). Trends in news media coverage of mental illness in the United States: 1995–2014. Health Aff..

[6] Kunitoh N., Suzuki W. (2015). Portrayal of mental illness in Japanese newspapers, 2001-2014. Act. Nerv. Super. Rediviva.

[7] Whitley R., Berry S. (2013). Trends in newspaper coverage of mental illness in Canada: 2005–2010. Can. J. Psychiatry.

[8] Owen P.R. (2012). Portrayals of schizophrenia by entertainment media: A content analysis of contemporary movies. Psychiatr. Serv..

[9] Domino G. (1983). Impact of the film,“One Flew Over the Cuckoo’s Nest,” on attitudes towards mental illness. Psychol. Rep..

[10] Perciful M.S., Meyer C. (2017). The impact of films on viewer attitudes towards people with schizophrenia. Curr. Psychol..

[11] Thonon B., Pletinx A., Grandjean A., Billieux J., Larøi F. (2016). The effects of a documentary film about schizophrenia on cognitive, affective and behavioural aspects of stigmatisation. J. Behav. Ther. Exp. Psy..

[12] Griffiths K.M., Carron-Arthur B., Parsons A., Reid R. (2014). Effectiveness of programs for reducing the stigma associated with mental disorders. A meta-analysis of randomized controlled trials. World Psychiatry.

[13] Corrigan P.W., Morris S.B., Michaels P.J., Rafacz J.D., Rüsch N. (2012). Challenging the public stigma of mental illness: A meta-analysis of outcome studies. Psychiatr. Serv..

[14] Clement S., Lassman F., Barley E., Evans-Lacko S., Williams P., Yamaguchi S., Slade M., Rüsch N., Thornicroft G. (2013). Mass media interventions for reducing mental health-related stigma. Cochrane Db. Syst. Rev..

[15] Couture S., Penn D. (2003). Interpersonal contact and the stigma of mental illness: A review of the literature. J. Ment. Health.

[16] Corrigan P.W., Penn D.L. (1999). Lessons from social psychology on discrediting psychiatric stigma. Am. Psychol..

[17] Allport G.W. (1954). The Nature of Prejudice.

[18] Corrigan P.W., Larson J., Sells M., Niessen N., Watson A.C. (2007). Will Filmed Presentations of Education and Contact Diminish Mental Illness Stigma?. Community Ment. Health J..

[19] Corrigan P.W., River L.P., Lundin R.K., Penn D.L., Uphoff-Wasowski K., Campion J., Mathisen J., Gagnon C., Bergman M., Goldstein H. (2001). Three strategies for changing attributions about severe mental illness. Schizophrenia Bull..

[20] Finkelstein J., Lapshin O., Wasserman E. (2008). Randomized study of different anti-stigma media. Patient Educ. Couns..

[21] Wahl O.F., Lefkowits J.Y. (1989). Impact of a television film on attitudes toward mental illness. Am. J. Community Psychol..

[22] Ritterfeld U., Jin S.-A. (2006). Addressing media stigma for people experiencing mental illness using an entertainment-education strategy. J. Health Psychol..

[23] Scarf D., Zimmerman H., Winter T., Boden H., Graham S., Riordan B.C., Hunter J.A. (2020). Association of viewing the filmes Joker or Terminator: Dark Fate with prejudice toward individuals with mental illness. JAMA Netw. Open.

[24] Kenny A., Bizumic B., Griffiths K.M. (2018). The Prejudice towards People with Mental Illness (PPMI) scale: Structure and validity. BMC Psychiatry.

[25] Knettel B.A. (2019). Attribution through the layperson’s lens: Development and preliminary validation of an inclusive, international measure of beliefs about the causes of mental illness. J. Person. Assess..

[26] Bizumic B., Duckitt J. (2018). Investigating right wing authoritarianism with a very short authoritarianism scale. J. Soc. Political Psychol..

[27] Ho A.K., Sidanius J., Kteily N., Sheehy-Skeffington J., Pratto F., Henkel K.E., Foels R., Stewart A.L. (2015). The nature of social dominance orientation: Theorizing and measuring preferences for intergroup inequality using the new SDO₇ scale. J. Pers. Soc. Psychol..

[28] Davis M.H. (1980). A multidimensional approach to individual differences in empathy. JSAS Cat. Selected Docs. Psychol..

[29] Lucas T., Zhdanova L., Alexander S. (2011). Procedural and distributive justice beliefs for self and others: Assessment of a four-factor individual differences model. J. Individ. Dif..

[30] Corrigan P.W., Rowan D., Green A., Lundin R., River P., Uphoff-Wasowski K., White K., Kubiak M.A. (2002). Challenging two mental illness stigmas: Personal responsibility and dangerousness. Schizophrenia Bull..

[31] Corrigan P.W., Shapiro J.R. (2010). Measuring the impact of programs that challenge the public stigma of mental illness. Clin. Psychol. Rev..

[32] Fischer D.G., Fick C. (1993). Measuring social desirability: Short forms of the Marlowe-Crowne Social Desirability Scale. Educ. Psychol. Meas..

